# Single layer centrifugation-selected boar spermatozoa are capable of fertilization *in vitro*

**DOI:** 10.1186/1751-0147-55-20

**Published:** 2013-03-05

**Authors:** Ylva Cecilia Björnsdotter Sjunnesson, Jane Margaret Morrell, Raquel González

**Affiliations:** 1 Department of Clinical Sciences, Division of Reproduction, Swedish University of Agricultural Sciences, P.O. Box 7054, Uppsala, SE 750 07, Sweden; 2 Centre for Reproductive Biology in Uppsala, Swedish University of Agricultural Sciences, P.O. Box 7054, Uppsala, SE 750 07, Sweden

**Keywords:** IVF, Pig, Semen, Sperm selection

## Abstract

**Background:**

Good quality spermatozoa are important to achieve fertilization, viable embryos and offspring. Single Layer Centrifugation (SLC) through a colloid (Androcoll-P) selects good quality spermatozoa. However, it has not been established previously whether porcine spermatozoa selected by this method maintain their fertility.

**Methods:**

The semen was prepared either by SLC or by standard centrifugation (control) and used for *in vitro* fertilization (IVF) at oocyte:spermatozoa ratios of 1:50; 1:100 and 1:300 (or 4 x 10^3^, 8 x 10^3^ and 24 x 10^3^ spermatozoa/ml) to evaluate their subsequent ability to generate blastocysts. In addition, sperm motility was assessed by computer assisted sperm motility analysis.

**Results:**

Total and progressive motility were significantly higher in sperm samples prepared by SLC compared to uncentrifuged samples. Sperm binding ability, polyspermy, cleavage and blastocyst rates were affected by the oocyte:sperm ratio, but not by sperm treatment.

**Conclusion:**

The use of SLC does not adversely affect the *in vitro* fertilizing and embryo-generating ability of the selected spermatozoa compared to their unselected counterparts, but further modifications in the IVF conditions would be needed to improve the monospermy in IVF systems. Since SLC did not appear to have a negative effect on sperm fertilizing ability, and may in fact select for spermatozoa with a greater potential for fertilization, an *in vivo* trial to determine the usefulness of this sperm preparation technique prior to artificial insemination is warranted.

## Background

During natural mating, physiological selection of suitable spermatozoa takes place in the female reproductive tract [1]. The availability of good quality spermatozoa is important to achieve successful fertilization, viable embryos and healthy offspring. Therefore, in an attempt to select the best spermatozoa in an ejaculate, several *in vitro* strategies have been developed for processing spermatozoa for their use in assisted reproduction [2,3]. Among these methods, colloid centrifugation separates spermatozoa from seminal plasma and selects spermatozoa on the basis of sperm motility, morphology, viability and chromatin integrity [4].

Single Layer Centrifugation (SLC) has arisen as an alternative method to other colloid preparations, providing several technical advantages, such as ready-to-use species-specific formulations; homogeneity between batches of colloid; and the possibility of scaling up to process whole/large ejaculates (as it is the case of the boar and the stallion), which may facilitate its application for commercial semen preparation purposes [4,5].

The fertilizing ability of bull spermatozoa selected by SLC has been demonstrated using *in vitro* fertilization (IVF) [6] and the functionality of SLC-treated stallion sperm has been proven after artificial insemination, showing promising results even with low quality semen [7].

SLC through a species-specific colloid (Androcoll-P), has also been developed for use with boar semen, enabling spermatozoa with good motility [8] and morphology [9] to be selected, while maintaining sperm plasma membrane ultrastructure and integrity [10,11]. However, the boar spermadhesins PSP-I and PSP-II (porcine seminal plasma proteins 1 and II, respectively), some of the proteins present in the seminal plasma, are removed from the sperm surface by SLC [10]. They are involved in maintaining or inhibiting sperm motility, membrane integrity, mitochondrial activity, zona pellucida binding and penetration and, ultimately, affect the ability of the spermatozoa to fertilize [12-14]. In addition, SLC may remove cholesterol from the sperm plasma membrane during the process of colloid centrifugation, although the integrity of the sperm membrane seemed not to be altered [10]. It is unknown if these, and possibly other, inadvertent, alterations of boar spermatozoa may hamper the fertilizing ability of SLC-selected spermatozoa and, therefore, it is essential to examine whether the fertilizing ability of SLC-selected boar sperm is affected before attempting artificial insemination.

Thus, the aim of this study was to evaluate the fertilizing ability of boar sperm selected by SLC and the production of embryos using *in vitro* fertilization and embryo culture. The main findings of the present study were that sperm selected by SLC were able to fertilize oocytes and the proportion of embryos developing to blastocyst was similar to the embryo production in their unselected counterparts.

## Methods

All reagents were purchased from Sigma-Aldrich, Stockholm, Sweden unless otherwise stated.

### Semen collection

All experiments were approved by the Ethical Committee for Experimentation with Animals, Uppsala, Sweden. The same boar (Swedish Landrace of proven fertility) was used throughout the experiment, housed and fed according to Swedish husbandry standards [15]. Semen was collected using the gloved hand technique, with a filter to remove the bulbo-urethral gland secretion. The sperm-rich fraction was extended after collection with Beltsville Thawing Solution (BTS) containing 0.2 mg/ml gentamicin sulphate (005974-ZG863, IMV technologies, L’Aigle, France) 1:1 (v/v) at 35°C and stored for 1–2 days at 16°C.

### Oocyte collection and *in vitro* maturation

Ovaries from gilts were obtained at an abattoir, placed in 0.9% (w/v) NaCl solution at 30–35°C and transported to the laboratory within 3–4 h of collection. Follicles with a diameter of 3–8 mm were aspirated using an 18-gauge needle and a 5 ml syringe. Aspiration medium consisted of HEPES-buffered TCM 199 (M 2520) with 26 mM NaHCO_3_ (S 5761), 1 mM L-glutamine (G 8540), 0.5 mg/ml polyvinyl alcohol (P 8136), 50 μg/ml gentamicin (G 1264) and 20 U/ml heparin (H 3149). The oocytes were retrieved in the same medium without heparin using a stereo microscope. Oocytes with even, dark cytoplasm and more than two compact layers of cumulus cells were selected, washed twice in *in vitro* maturation medium (IVM) and placed in groups of 40–60 in a final volume of 500 μl of IVM. Oocytes were cultured at 38.5°C under 5% CO_2_ in air and maximum humidity for 46 h. The maturation medium consisted of TCM 199 (M 2154) with 1 mM L-glutamine, 1 mM sodium pyruvate (P 3662), 0.1 mM 2-mercaptoethanol (M 7522), 50 ng/ml epidermal growth factor (E 4127), 50 μg/ml gentamicin and 0.1% (w/v) BSA (A 3311). During the first 24 h of IVM, the medium was supplemented with 10 IU/ ml PMSG and 5 IU/ ml hCG (Suigonan Vet, Intervet Scandinavia, Skovlunde, Denmark) and 5 μl/ml ITS (recombinant human insulin, human transferrin, and sodium selenite, I 3146).

### Sperm preparation

The semen was prepared either by SLC or by standard centrifugation (control). The SLC was performed according to [8]. In brief, the extended semen was further diluted in BTS to approximately 1 × 10^8^ sperm cells/ml; 4.5 ml were carefully layered on top of 4 ml Androcoll-P in a 15 ml tube. The tube was centrifuged at 300 × *g* for 20 min at room temperature and the supernatant was removed. The sperm pellet was transferred to pre-gassed IVF medium (see below). An additional SLC-sperm preparation was done in parallel to provide a sample for computer assisted sperm motility analysis. The sperm pellet from the latter was resuspended in BTS containing BSA (5 mg/ml) to prevent the spermatozoa aggregating or sticking to the glass surfaces during motility assessment [8]. For the control group, sperm was prepared by two centrifugations in 4 ml of non-capacitating medium at 300 × *g* for 5 min at room temperature. Sperm non-capacitating medium consisted of 113 mM NaCl (S 5886), 5 mM KCl (P 5405), 6 mM D-glucose (G 6152), 1 mM KH_2_PO_4_ (P 5655), 1 mM MgSO_4_ · 7H_2_O (M 1880), 22 mM lactic acid (L 7900), 5 μg/ml phenol red (P 5530) and 50 μg/ml gentamicin. The resulting pellets from both preparation methods were diluted in 4 ml of IVF medium and kept at 38.5°C in 5% CO_2_ in air and maximum humidity for 15 min while sperm concentrations were determined using a NucleoCounter SP-100 (ChemoMetec, Allerød, Denmark). To reach the desired sperm concentrations and oocyte:sperm ratios, the sperm dilutions were further diluted in IVF medium.

### Computer assisted sperm motility analysis

Sperm motility was analyzed using a Mika Cell Motility Analyser (MTM Medical Technologies Montreux, Switzerland) after incubating the samples at 38°C for 30 min; only the total and progressively motile spermatozoa were recorded for this experiment. Motility was evaluated in BTS containing BSA (5 mg/ml). An aliquot (5μL) of the sample in a pre-warmed Makler counting chamber (Sefi Medical Instruments, Haifa, Israel) was analyzed using the following software settings: 32 frames per sequence, a minimum of 15 frames per object, minimum area for objects 25 pix, velocity limit for immobile objects = 10 μm/s, velocity limit for locally motile objects = 25 μm/s [16]. Motile spermatozoa were classified as linear (spermatozoa deviating less than 10% from a straight line), circular (spermatozoa moving in a circle with a radius less than 25 μm), and non-linear (spermatozoa falling into neither of the first two categories). At least 200 spermatozoa were analyzed for each sample.

### *In vitro* fertilization

After *in vitro* maturation, the oocytes were washed twice in IVF medium and randomly assigned to the different sperm treatments. Spermatozoa were prepared as indicated above and diluted in IVF medium to compare the following treatments: control (standard centrifugation) at 1:50 and 1:100 oocyte:sperm ratios or SLC-treated at 1:50; 1:100 or 1:300 (corresponding to 4 × 10^3^; 8 × 10^3^ or 24 × 10^3^ cells/ml, respectively). IVF medium consisted of 90 mM NaCl, 12 mM KCl, 0.5 mM NaH_2_PO_4_ (S 5011), 25 mM NaHCO_3_, 1 mM MgSO_4_ · 7H_2_O, 2 mM sodium pyruvate, 8 mM CaCl_2_ · 2H_2_O (C 7902), 6 mM lactic acid, 1 mM caffeine (C 0750), 5 μg/ml phenol red, 50 μg/ml gentamicin, and 0.6% (w/v) BSA. Since the number of available oocytes was different between batches, the groups of oocytes per treatment ranged from 40 to 49 depending on the experimental day. To keep the IVF conditions constant using the total number of oocytes available, approximately 12 μl of IVF medium per oocyte was used (500–612 μl IVF medium depending on the replicate). Therefore the number of oocytes per group and the final IVF volume were equal in each replicate. The oocyte:sperm ratio and the final sperm concentration were also constant between replicates to allow comparisons. Nine independent experiments were performed (1519 oocytes). Since three to five treatments could be tested per batch, the treatments were rotated throughout the experiments (always with control present, resulting in 6–8 replicates per group; Table 1). The gametes were incubated together at 38.5°C under 5% CO_2_ in air and maximum humidity for 24 h. Day 0 was defined as the day of fertilization.

**Table 1:** **Effect of sperm treated with colloids at different oocyte:sperm ratios on *in vitro *embryo development (mean ± SEM)**

**Treatment Ratio oocyte: spermatozoa**	**n**	**Spermatozoa bound to the ZP**	**Polyspermic oocytes (%)**	**Fertilization rate (%)**	**Cleavage rate (48 hpi)%**	**Blastocyst rate (day 7)%**
		**No. spermatozoa (over non-cleaved oocytes)**	**(over non-cleaved oocytes)**	**Over No. Presumptive zygotes**	**No. embryos/No. presumptive zygotes**	**No. blastocysts/No. presumptive zygotes**
**Control 1:50**	6	9.4 ± 6.5 ^a^	8.2 ± 3.0 ^a^	59.3 ± 4.4 ^a^	55.7 ± 4.0 ^a^	12.5 ± 2.0 ^a^
		(344/128)	(11/126)	(175/292)	(164/292)	(37/292)
**Control 1:100**	8	5.1 ± 0.6 ^a^	27.6 ± 6.2 ^b^	67.0 ± 5.1 ^ab^	55.1 ± 4.9 ^a^	17.7 ± 3.7 ^a^
		(758/145)	(37/141)	(215/317)	(174/317)	(55/317)
**SLC 1:50**	6	2.9 ± 0.6 ^a^	15.3 ± 5.4 ^a^	62.9 ± 5.5 ^a^	56.0 ± 4.2 ^a^	16.0 ± 4.1 ^a^
		(328/136)	(16/134)	(183/304)	(167/304)	(47/304)
**SLC 1:100**	8	6.7 ± 1.2 ^a^	36.4 ± 7.9 ^b^	73.5 ± 3.4 ^ab^	56.2 ± 3.2 ^a^	15.8 ± 2.2 ^a^
		(944/135)	(51/132)	(244/315)	(176/315)	(49/315)
**SLC 1:300**	7	20.5 ± 2.1 ^b^	66.5 ± 6.7 ^c^	78.4 ±2.8 ^b^	31.0 ± 4.3 ^b^	5.2 ± 1.4 ^b^
		(4118/194)	(133/194)	(224/291)	(91/291)	(15/291)

Different letters denote significant differences between treatments (at least P < 0.05). The number of replicates where each treatment was included is indicated (n). ZP: zona pellucida.

### *In vitro* culture and blastocyst assessment

The oocytes (presumptive zygotes) were transferred to 750 μl of NCSU-23 [17] supplemented with 10 mM HEPES (H-6147), 50 μg/ml insulin (I-6634) and 25 μg/ml gentamicin. Any remaining cumulus cells and excess spermatozoa were removed by vortexing for 1 min. Some oocytes were not recovered during this process, presumably due to adhesion to plastic and/or ruptured zona pellucida; therefore, data are expressed over the total number of retrieved and subsequently cultured presumptive zygotes. After vortexing, presumptive zygotes were transferred to 500 μl of *in vitro* culture medium (PZM-3) [18]. The numbers of early cleaved zygotes at 24 h after fertilization and total cleavage rates (48 h post insemination, hpi) were assessed in HEPES-NCSU-23. At this point, non-cleaved oocytes were removed and stained. Fertilization ratios were calculated retrospectively and included the percentage of non-cleaved oocytes with ≥ 2 pronuclei-like structures (thus, including polyspermic oocytes) that were arrested and were not able to cleave, plus the embryos found at 48 hpi. The embryos were transferred to fresh PZM-3 in groups of 35–45 and covered with 300 μl of mineral oil (M 8410). Culture was maintained up to day 7 at 38.5°C in a mixture of 5% CO_2_, 5% O_2_ and 90% N_2_ and maximum humidity. On day 7 after fertilization, blastocyst rates were evaluated and the blastocysts were graded according to the International Embryo Transfer Society classification (scored one to four where one is the best grade) [19].

### Nuclear staining

Non-cleaved oocytes and blastocysts were fixed in 2% paraformaldehyde overnight at 4°C and stained with 4.45 μM Hoechst 33342 (B 2261) for 20 min at room temperature before being mounted in Vectashield (Vector laboratories, Inc. Burlinggame, CA, USA) on glass slides and assessed using an epifluoresent microscope (Leitz GmbH Dialux 20, Wetzlar, Germany). The non-cleaved oocytes were assessed for polyspermy (more than two pronuclei-like objects per oocyte) and number of accessory spermatozoa bound to the zona pellucida (ZP). The number of nuclei in the blastocysts was counted.

### Statistical analysis

Statistical analyses were performed using Statistica (Statsoft, Inc. 2001. STATISTICA, data analysis software system, version 6; http://www.statsoft.com). The number of spermatozoa bound to the ZP, the percentage of polyspermic oocytes, fertilization, cleavage and blastocyst rates were analyzed using main-effects ANOVA where sperm treatment (control or SLC) and oocyte:spermatozoa ratios (1:50, 1:100 or 1:300) were considered as independent variables. Before the analysis, normal distribution of data and residuals were tested using Shapiro-Wilk’s test. Homogeneity of variances was evaluated using Levene’s test. Dependent variables expressed as percentages were arcsine-transformed and the number of spermatozoa attached to the ZP was log-transformed to satisfy parametric assumptions. When significant differences were found, comparison between treatments was made using the *post hoc* Tukey test. The blastocyst nuclei number and quality scoring on day 7 were analyzed using Kruskal-Wallis ANOVA. In addition, total and progressive sperm motility in SLC or uncentrifuged samples were evaluated using a t-test for dependent samples. Results are presented as mean ± S.E.M, with P < 0.05 being considered significant.

## Results

Both the total motility and progressive motility were significantly higher in SLC-sperm samples than in uncentrifuged semen (P < 0.05) (Table 2). The number of spermatozoa bound to the ZP was not affected by sperm treatment, but there was an effect of the oocyte:sperm ratio (F_2,31_ = 14; P < 0.001). A significantly higher number of spermatozoa were bound in the 1:300 than 1:50 and 1:100 groups (Table 1). Sperm treatment did not affect the percentage of polyspermic oocytes, but polyspermy was influenced by sperm concentration (F_2,31_ = 16.9; P < 0.001). Increasing oocyte:sperm ratios resulted in increased polyspermy and fertilization rates (P < 0.05) (Table 1). Early cleavage rate was similar in all groups, ranging from 6.0 ± 2.0 to 7.1 ± 1.4% (P > 0.05). The total cleavage rate was not affected by sperm treatment, but was affected by sperm concentration (F_2,31_ = 12.7; P < 0.001), being significantly lower in the SLC 1:300 group (Table 1). Blastocyst yield was not affected by the sperm preparation method but was affected by sperm:oocyte ratios (F_2,31_ = 7.4; P < 0.003), with the SLC 1:300 group having a significantly lower blastocyst rate (Table 1). Blastocyst quality grade and number of blastomeres did not differ (Table 3).

**Table 2:** Sperm motility

**Treatment**	**n**	**Total motility% (CI)**	**Progressive motility% (CI)**
**Uncentrifuged**	9	71 ± 7 ^a^	69 ± 6 ^a^
		(54 to 87)	(54 to 83)
**SLC**	9	91 ± 2 ^b^	79 ± 5 ^b^
		(87 to 94)	(69 to 89)

Mean ± SEM and 95% confidence interval (CI).

Different letters denote significant differences between treatments (P < 0.05). The number of replicates where each treatment was analyzed is indicated (n).

**Table 3:** Blastocyst nuclei counts and blastocyst quality (mean ± SEM)

**Treatment Ratio oocyte: spermatozoa**	**n**	**Blastocyst cell number**	**n**	**Blastocyst quality grade**
**Control 1:50**	32	42.7 ± 3.3	35	2.07 ± 0.11
**Control 1:100**	44	39.2 ± 2.4	50	1.94 ± 0.10
**SLC 1:50**	47	41.4 ± 2.2	45	1.79 ± 0.08
**SLC 1:100**	36	41.7 ± 2.3	39	2.19 ± 0.13
**SLC 1:300**	10	41.3 ± 4.2	13	2.00 ± 0.24

There were no significant differences between groups. The number of blastocysts analyzed is indicated (n). Blastocyst quality grade range was 1 to 4, where grade 1 is the best grade.

## Discussion

This study has allowed us to evaluate the fertilizating ability of boar sperm selected by SLC using *in vitro* matured oocytes. The SLC treatment did not negatively affect the ability of boar spermatozoa to undergo fertilization and the embryos generated were able to develop to the blastocyst stage in the same proportion as the embryos produced in the control group.

SLC through Androcoll-P selects good quality boar spermatozoa [8,9]. However, previous attempts in our laboratory using IVF with these spermatozoa resulted in polyspermic zygotes, precluding embryo development (Sjunnesson YCB, González R and Morrell JM, unpublished observations). It is likely that the increased motility in the SLC samples, and probably improvements in other variables of sperm quality such as morphology and viability [8,9], may have accounted for the increased polyspermy caused by the use of Androcoll-P (Sjunnesson YCB, González R and Morrell JM, unpublished observations). This is in agreement with previous studies by others using colloid-prepared spermatozoa for IVF in the boar. Sperm treated by centrifugation through discontinuous Percoll gradient displayed higher motility and sperm binding to the ZP, and penetration rates were also enhanced, which resulted in an increase in polyspermy [20,21]. Since colloid-selected spermatozoa seemed to have an increased ability to fertilize [20,21] or at least improved sperm quality [8,9], the ability of SLC-prepared spermatozoa to fertilize *in vitro* matured oocytes with subsequent development to the blastocyst stage was assessed in the present study with lower oocyte:sperm ratios than previously used. No negative effects of SLC-treated spermatozoa on *in vitro* parameters such as cleavage, blastocyst rate or blastocyst quality compared to controls were observed, in agreement with the results reported using SLC-treated bull spermatozoa [6]. However, the establishment of pregnancy and litter size after embryo transfer of these blastocysts, as well as the *in vivo* fertilization ability of SLC-selected spermatozoa, has yet to be ascertained.

Polyspermy, caused by simultaneous penetration by several spermatozoa [22,23], occurs *in vivo* and *in vitro* in the pig [24], and could be a cause of embryonic loss [22]. In the present study, few embryos developed when oocyte:sperm ratios over 1:100 were used in the SLC-group. Surprisingly, although the 1:300 ratio was not included in the control, the commonly used ratios in the control treatment for this particular sperm donor (between 1:1250 to 1:3000), resulted in a cleavage rate of around 50% and blastocyst rate above 15% (Sjunnesson YCB, González R, Morrell JM, unpublished observations). The lower cleavage and blastocyst rates consistently observed in the SLC 1:300 group were most probably due to the significantly higher polyspermy seen in this group. In agreement with our results, spermatozoa treated with Percoll had faster oocyte penetration and higher blastocyst yield, and a reduced incubation time of 2 h of gamete interaction was found to be sufficient, since longer incubations caused increased polyspermic rates [20]. In the latter study, an oocyte:spermatozoa ratio of approximately 1:715–1:830 (25 × 10^4^ sperm cells/ml) was used for up to 6 h of gamete co-incubation, in comparison to the longer incubation time of gametes (24 h) in the present study, which may lead to a higher incidence of polyspermy when sperm concentration was increased in the SLC-group. The percentage of acrosome-reacted spermatozoa was greater after treatment with Percoll [20]. It is unknown if the acrosome reaction and capacitation status of boar spermatozoa are changed in SLC-treated sperm, although these parameters are currently under investigation. Furthermore, it would be interesting to determine these parameters using shorter incubation times. This would allow evaluation of the fertilizing ability of SLC-treated sperm versus control samples and determination of the optimal time of gamete incubation to increase the efficiency of the IVF system using spermatozoa prepared by SLC.

Our results demonstrate that the function of boar SLC-selected sperm is not hampered in IVF. However, the advantages of SLC in IVF for boars which already have high sperm quality may be difficult to evaluate because of the problem of polyspermy. Nonetheless, SLC could be very useful in genetically valuable males with reduced semen quality or in non-commercial breeds that present lower male selection for breeding purposes and, therefore, a wider range in semen quality.

In comparison to other colloid methods, such as density gradient centrifugation, the use of SLC present advantages for boar semen due to its simplicity of preparation, species-specific formulations and the possibility of processing large volume of ejaculates [8]. In addition, SLC could be useful for selecting good quality spermatozoa after freezing [25,26] and sex sorting where numbers of viable spermatozoa can be low [27,28]. SLC could also be a useful tool in porcine intracytoplasmic sperm injection [29] where the aspect of polyspermy is irrelevant. Moreover, SLC has shown promise in removing or significantly reducing bacteria and viruses in pig semen [5,30] and, as such, may have an important role to play in biosecurity in the future and may help to reduce the use of antibiotics in semen extenders.

## Conclusion

In summary, SLC can select boar sperm without affecting their functionality, as demonstrated in the IVF system. Boar semen selected by means of SLC support embryo development *in vitro* at the same rates as their untreated counterparts. Since SLC-samples seemed to have an increased fertility potential, boar-related adjustments to the IVF conditions and probably shortening the interaction period between gametes will be needed to avoid an increased incidence of polyspermy and improve the efficiency of the IVF system. These results could be important for the future use of SLC-prepared sperm in artificial insemination trials.

## Abbreviations

ANOVA: Analysis of variance; BSA: Bovine serum albumin; BTS: Beltsville thawing solution; hCG: Human chorionic gonatotropin; HEPES: 4-(2-Hydroxyethyl)-1-piperazineethanesulfonic acid; Hpi: Hours post insemination; ITS: Insulin, transferrin, and sodium selenite; IVF: *In vitro* fertilization; IVM: *In vitro* maturation; NCSU: North Carolina State University; PSP-I: PSP-II, Porcine seminal plasma proteins I and II; PMSG: Pregnant mare’s serum gonatotropin; PZM: Porcine zygote medium; SEM: Standard error of the mean; SLC: Single layer centrifugation; ZP: Zona pellucida.

## Competing interests

The authors declare there are no competing interests.

## Authors’ contributions

YCBS discussed the proposal, approved the final plan, prepared the oocytes for IVF and culture of embryos as well as analyses of oocytes and blastocysts and did the major writing of the article. JMM discussed the proposal, approved the final plan, performed the SLC and motility analysis, assisted with preparing the oocytes for IVF, assisted with writing the article and has approved the final draft. RG discussed the proposal, approved the final plan, prepared the oocytes for IVF and culture of embryos as well as analyses of oocytes and blastocysts, performed all statistical analyses and assisted with writing the article and has approved the final draft. All authors read and approved the final manuscript.
